# Case report: Application of color Doppler sonography for the assessment of pulmonary consolidations in a dog

**DOI:** 10.3389/fvets.2023.1275929

**Published:** 2023-12-08

**Authors:** Katarzyna Kraszewska, Michał Gajewski, Søren R. Boysen

**Affiliations:** ^1^Vetcardia Veterinary Clinic, Warsaw, Poland; ^2^Faculty of Veterinary Medicine, University of Calgary, Calgary, AB, Canada

**Keywords:** dog, lung ultrasonography (LUS), thromboembolic disease, consolidation, color Doppler

## Abstract

A 1,5-year-old male Maltipoo was presented to the emergency service for dyspnea, weakness, and cough. An echocardiographic examination showed evidence of pulmonary hypertension with a McConnell sign. Lung ultrasound, including color Doppler, was performed and identified two distinct populations of lung consolidation. Color Doppler analysis of the first type of consolidation showed the absence of blood flow within regions of the consolidation and flow amputation. These findings were consistent with the “*vascular sign*” reported in human medicine and prompted consideration of pulmonary thromboembolism as a differential diagnosis. In the second type of consolidation, color Doppler identified blood flow within the pulmonary vessels of the consolidated lung, forming a “branching tree-like” pattern that followed the anatomical course of the pulmonary vasculature. These findings suggested that blood flow was preserved within the pulmonary vasculature of the consolidated lung and prompted consideration of inflammatory causes of pulmonary pathology. On recheck 6 days later, recanalization of the first type of consolidation was identified with color Doppler. The case was followed serially once a month for 5 months with LUS, which showed continued improvement. Based on a positive fecal Baermann test, a final diagnosis of *Angiostrongylus vasorum* was made. New or Unique information Provided—this is the first report of color Doppler LUS being used to characterize and help differentiate the cause of lung consolidation in dogs.

## Introduction

In human medicine, color Doppler analysis is one component of lung ultrasound (LUS) that can help differentiate thromboembolic from inflammatory pulmonary lesions (1–4). The absence of blood flow within regions of lung consolidation and the so-called “*vascular sign*” are consistent with thromboembolic pulmonary disease and are believed to result from the occlusion of a vessel by embolic material (4). By contrast, the identification of blood flow and a “*tree-like*” vascular pattern within regions of lung consolidation are more consistent with inflammatory causes of pulmonary disease (2, 4–6). Pulmonary thromboembolism (PTE) is challenging to diagnose in veterinary patients, as clinical signs are non-specific and readily available diagnostics, including thoracic radiographs, are neither sensitive nor specific. The reference standard, selective computed tomography pulmonary angiography (CTPA), requires specialized equipment that is not readily available and is restricted by the need to transport patients and the requirement for general anesthesia, sedation, and/or physical restraint (7). By contrast, ultrasound has become commonplace in many veterinary clinics and is often used to assess the lungs of small animal patients in respiratory distress (8). Lung ultrasound is minimally invasive, well tolerated, and can be performed without the need for sedation, analgesia, or specific patient positioning, which minimizes the risk of patient deterioration when evaluating patients in respiratory distress. Furthermore, LUS has been used to support a diagnosis of PTE in dogs through the identification of “wedge signs” (9). Unfortunately, “wedge signs,” identified in isolation, or located ventrally, are not considered sensitive or specific for PTE (4, 10). To the authors’ knowledge, color Doppler sonography of the lung has not been used clinically in small animal patients. This case report describes how color Doppler analysis of consolidated pulmonary parenchyma can be used to characterize and help differentiate pulmonary pathology based on the preservation or lack of blood flow.

## Case description

A 1.5-year-old male Maltipoo was referred to the emergency service for dyspnea, weakness, cough, and anorexia of 3 days duration. On physical examination, the dog was tachypneic (40 breaths/min) with marked respiratory effort, had local cranio-dorsal crackles auscultated bilaterally, and had a rectal body temperature of 36.6°C. A right apical systolic heart murmur (grade 1/6) was noted on cardiac auscultation. Thoracic point-of-care ultrasound performed by the attending emergency clinician identified diffuse B-lines caudodorsally. Three-view thoracic radiographs (TXRs) (ventrodorsal, dorsoventral, and right lateral), taken without sedation, revealed a bilateral bronchial pattern in the caudal lung regions and a cranial alveolar pattern, which was more prominent on the right (Supplementary Figure S1). The vertebral heart score was 9.5 and considered normal for the standard-shaped thorax of the dog (11). A full in-house complete blood count (CBC) and serum chemistry panel showed neutrophilia (20.63 G/L, reference values 6–12 G/L) with all other measured blood parameters within reference limits. Following initial diagnostic testing, the patient received supplemental oxygen via an oxygen case, furosemide (2 mg/kg), antibiotic therapy (enrofloxacin 5 mg/kg SID), and theophylline (10 mg/kg BID) for suspected aspiration pneumonia and possible small airway disease based on the cranial ventral alveolar and dorsal bronchial patterns seen on TXR, respectively.

Due to the presence of a heart murmur and the detection of increased B-lines, an echocardiogram was performed the following day by an experienced clinician with >10 years of experience in cardiology (KK) using a GE VIVID IQ ultrasound machine with two phrased array probes of 12 and 6 MHz frequency. Echocardiography was performed with the patient in a standing position due to persistent respiratory distress, which had worsened relative to the time of presentation. The higher-frequency probe was used to assess the cardiac structures in high-resolution detail with a ready-made “neonate” preset with a depth at 6 cm and focus position on 3 cm. The low-frequency probe, because it has better-quality continuous-wave Doppler, was used to measure the velocity of any tricuspid and pulmonic valve insufficiency. The velocity was automatically calculated from m/s to mmHg using the Bernoulli equation (*p* = 4 × V^2^). The echocardiographic evaluation revealed a scant amount of pericardial effusion (PE) at the cardiac apex of the right ventricle, severe eccentric and concentric hypertrophy of the right ventricle in diastole (RVDd), and systole (RVDs) (Supplementary Figure S2), paradoxical motion of the interventricular septum, and tricuspid valve regurgitation (TVR) with a high-velocity regurgitant jet. A diagnosis of pulmonary hypertension was made based on objective cardiac measurements (Table 1) in conjunction with the recent pulmonary hypertension guidelines (12, 14–17). On the left apical view, akinesis of the right ventricle with apical sparing, also known as McConnell’s sign, was observed, which has been reported to be secondary to acute pulmonary thromboembolism (PTE) (18, 19). An agitated saline contrast study revealed no evidence of intracardiac or extracardiac right-to-left shunting.

**Table 1:** Cutoff and reference values for selected echocardiographic parameters of PHT for the prediction of peak tricuspid regurgitant systolic pressure gradient >50 mmHg at admission, after 6 days, and after 45 days (12, 13).

	View	On presentation	6-day recheck	45-day recheck
AccT/ET PA (cutoff value <0.30)	R-parasternal	0.23	0.3	0.34
MPA/Ao (cutoff value >1.04)	R-parasternal	1.28	1.18	1.07
PAR (m/s) (cutoff value >2.5 m/s)	R-parasternal	3.03	2.76	−
PE	L-apical	+	−	−
Raa index (cm^2^/m^2^) (reference value 4.1–10.1 cm^2^/m^2^)	R-parasternal	13.92	12.1	9.56
rPADI (%) (cutoff value <29.5%)	R-parasternal	9	13.6	31,1
RVDd (cm) M-mode	R-parasternal	1.85	1.33	0.87
TVR (m/s) (cutoff value <3.4 m/s)	L-apical	5.68	4.7	−

AccT, acceleration time; ET, ejection time; L-apical, left apical four-chamber view; MPA, main pulmonary artery; PA, pulmonary artery; PAR, pulmonary artery regurgitation; PE, pericardial effusion; Raa index, right atrial area index; rPADI, right pulmonary artery distensibility index; R-parasternal, right parasternal short axis view; RVDd, right ventricular internal diameter in diastole; TVR, tricuspid valve regurgitation.

Given there was no obvious cardiogenic cause for pulmonary hypertension and TXRs suggested pulmonary disease, a lung ultrasound examination was performed by the same operator (KK, with over 5 years of experience in performing LUS with advanced training from human lung ultrasound experts) using a GE VIVID IQ and a multifrequency linear probe (6–12 MHz). Two presets were used during the examination. For the identification and characterization of lung surface artifacts, the pleural line, and lung sliding, a special “lung preset” was utilized. This preset has harmonics turned off, uses the lowest frequency of the probe (6 MHz) with persistence turned to zero, has a focal position set at the level of the pleural line, and uses an increased time gain compensation (TGC) at the distal (far) field of the screen. Such settings create a “coarser” picture (20, 21). Color Doppler analysis of the consolidations was performed on this preset, with the Nyquist limit set at the level of 0.7 m/s. For better visualization of consolidations, the preset was switched to a default “thyroid” preset. “Thyroid” preset has harmonics turned on, uses a higher dynamic range, and has spatial compound imaging turned on, resulting in a “smoother” image for a better depiction of the consolidation; the focus was set at the center of the consolidation.

**Figure 1:**
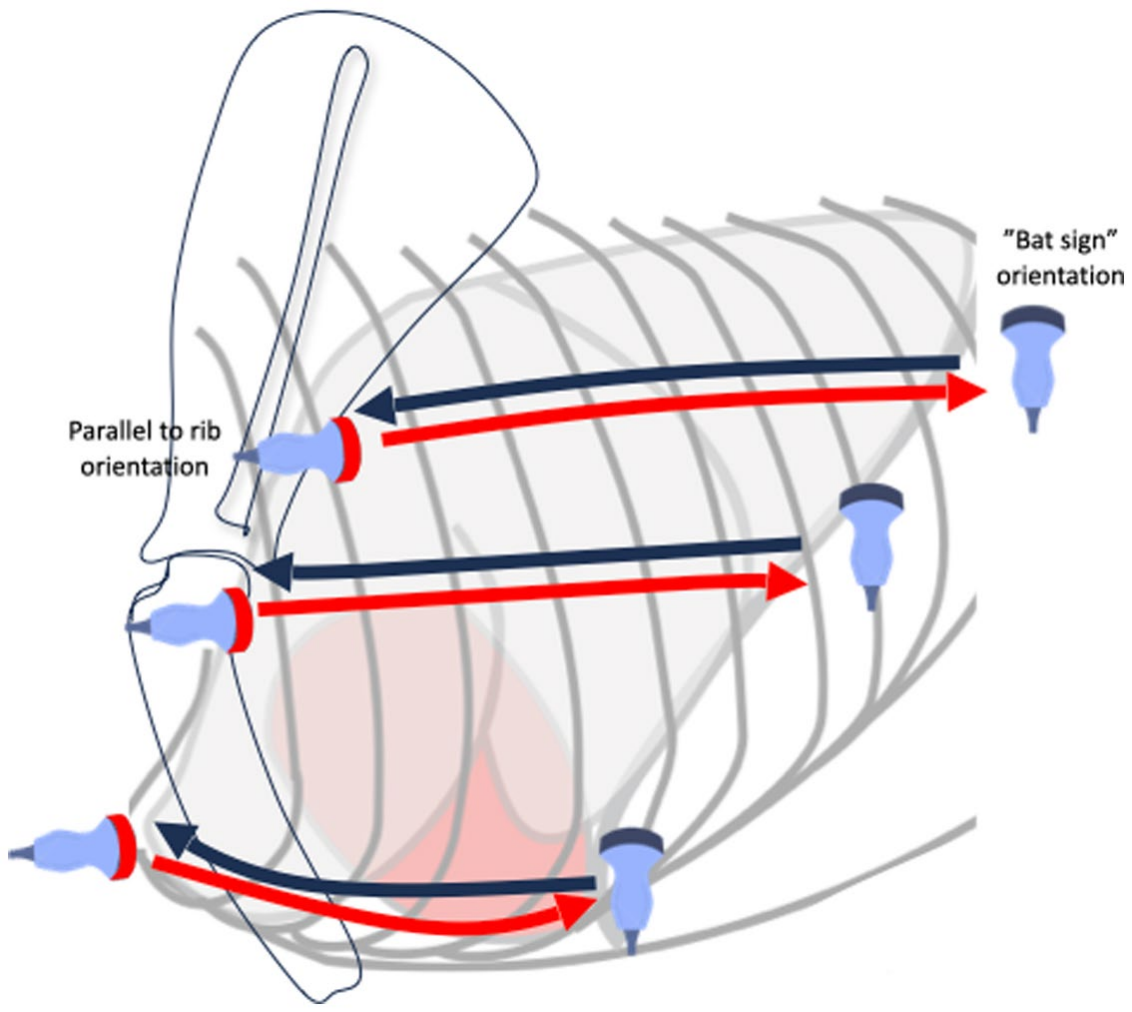
Diagram showing lung ultrasound examination protocol.

The dog was examined in a standing position. Alcohol and gel were used as coupling agents. The lung ultrasound examination followed the VetLus protocol Figure 1 (22), created by the authors (K.K. and M.G.). It utilizes a horizontal sliding technique with the transducer placed at three different vertical locations on each side of the thorax at a level in line with: (1) the middle of the scapula below the rib heads and epaxial muscles (dorsal line); (2) the shoulder joint (at the level of the heart base, middle line); and (3) just dorsal to sternebrae (ventral line). The starting orientation of the probe is in the longitudinal plane relative to the long axis of the body (frontal plane, perpendicular to the ribs). First, the transducer was slid along the dorsal line from cranial to caudal to identify the bat sign and to assess as much of the lung surface as possible. Following this initial scan, the probe was rotated 90 degrees into a transverse plane; probe orientation is therefore longitudinal relative to the ribs, and the intercostal spaces are assessed by moving the probe from a caudal to cranial direction along the same dorsal line. This gives an image of a continuous pleural line without rib shadows, which were visible in the frontal plane. The same scanning technique with sliding the probe in the frontal plane followed by the transverse plane was repeated at the second- and third-line locations (i.e., the shoulder and parasternal line). The technique was then repeated on the opposite hemithorax.

Whenever the operator encountered vertical artifacts or consolidations on LUS examination, a cineloop was recorded for further characterization. The characteristics of every lung consolidation included their size, shape, margins, echogenicity, and additional features, which can be divided into parenchymal (presence of bronchograms), vascular (using color Doppler sonography), and pleural (presence or absence of lung sliding, effusion, and pleural line irregularities) criteria, as defined by recent human lung ultrasound guidelines (4, 23). Two populations of consolidation were subsequently identified based on their shape and vascular characteristics: (1) irregular-shaped “shred”-type consolidations with visible vascular blood flow and (2) triangular or basket-shaped consolidations without blood flow and/or with a “*vascular sign*” (4, 9, 10, 24, 25). The characteristics of these two types of consolidations are summarized in Table 2.

**Table 2:** Characteristics of two types of consolidations identified on lung ultrasound together with their potential etiology.

	Shape	Margins	Parenchymal criteria	Vascular criteria	Pleural criteria	Suspected etiology
Type 1	Irregular, “shred” type	Serrated, irregular	Air bronchograms, rare fluid bronchograms	Anatomical, tree-like vasculature	Locally decreased or absent lung sliding	Pneumonia
Type 2	Triangular or basket-shaped	Smooth	No bronchograms	No flow and/or “vascular sign”	Small amount of pleural effusion, decreased lung sliding	PTE

Irregularly shaped consolidations were predominantly restricted to the caudodorsal lung lobes and had visible static and dynamic air bronchograms (4, 10, 24) (Supplementary Figure S3). At the periphery of some consolidated regions, a fluid bronchogram was observed. Comet-tail artifacts, extending from the far edge of these consolidations, also referred to as C-lines, were also noted (24, 26). Color Doppler imaging identified perfused vessels within these consolidations, indicating that the vascular system was intact and followed the anatomical course of blood vessels, thus taking on the shape of a tree (Supplementary Figure S3) (3, 27). Lung sliding was locally absent at the thoracic wall-to-consolidation interface (proximal surface of the consolidation).

**Figure 2:**
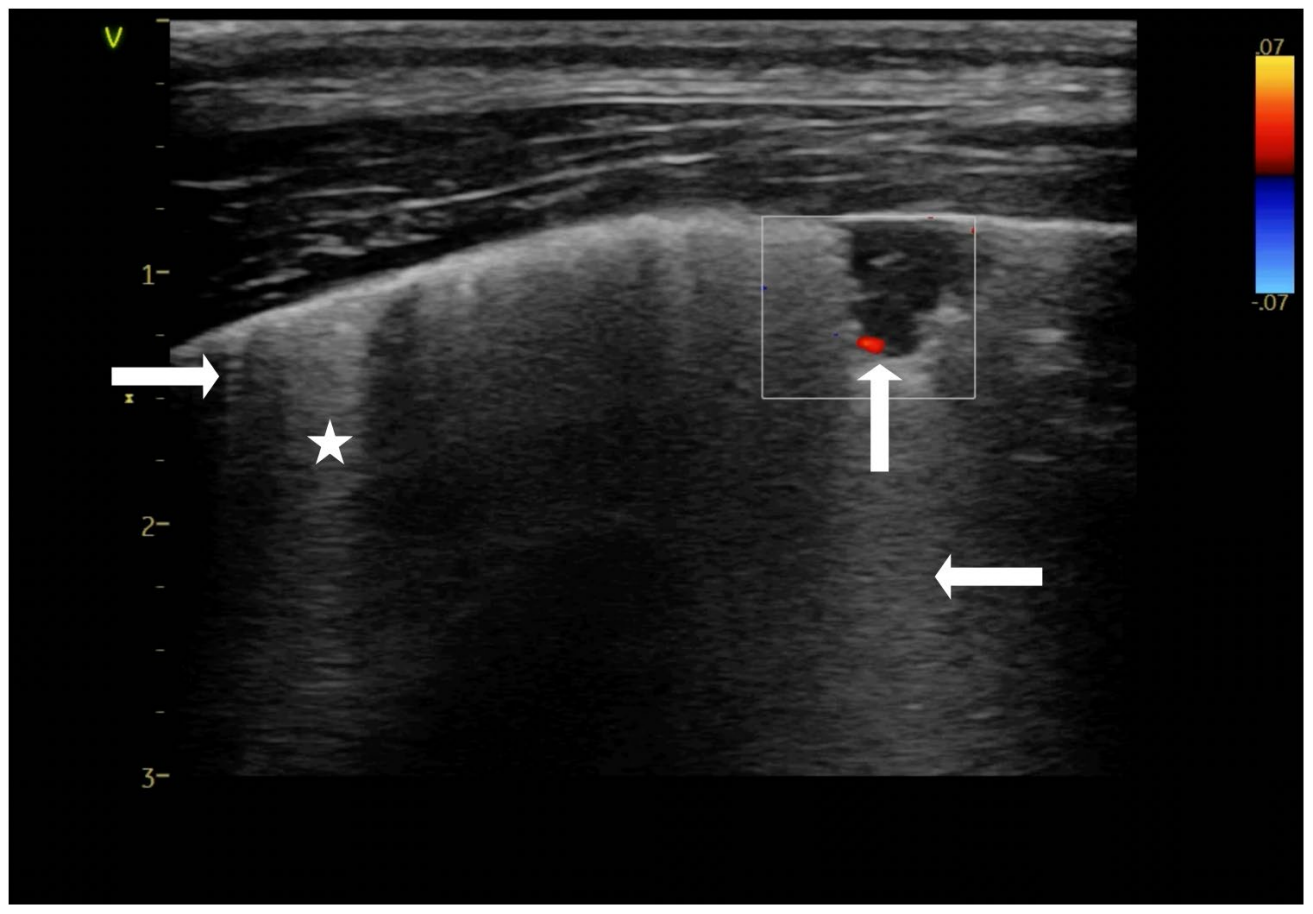
Color doppler still image of a triangular shaped consolidation within the lung referred to as a “wedge sign”. An absence of blood flow within the consolidated region is visible, giving the impression the blood flow is “amputated” (↑), which is referred to as the “vascular sign”. A comet-tail artifact (C-line) extends from the lower edge of the consolidation (←). Short artifacts originating at the pleural line (→) are also visible, referred to as I-lines. Finally, hyperechoic vertical artifacts, believed to be B-lines are also visible (*). To confirm these are B-lines the depth should be extended (10-15 cm) to ensure these vertical white artifacts continue to reach the far field. Performed at presentation. Thyroid presets.

Triangular-shaped lesions, with the base of the triangle facing the pleural line, were observed in the cranial lung lobes. In addition to triangle-shaped lesions, an occasional basket-shaped lesion was identified, appearing as a triangle with the “tip” chopped off (blunted wedge). Both triangular-shaped and basket-shaped lesions were classified as “wedge signs” due to shared LUS and color Doppler imaging characteristics and their resemblance to the anatomical shape of a pulmonary lobule (Figure 2) (9). The length of consolidations from the apex to the base of the “wedge sign” (diagonal of the triangle) varied between 3 and 20 mm, with comet-tail artifacts (C-lines) extending from the far edge of the consolidation. Adjacent to “wedge signs,” B-lines were noted. Spared lung areas (normal aerated lung) were identified in addition to consolidated lung regions and B-lines, as well as short, needle-like vertical artifacts, referred to as I-lines (Figure 2) (28). The parenchymal characteristics of these lesions included hypoechoic consolidation with the absence of bronchograms. A trace amount of pleural fluid between the “wedge sign” and thoracic wall could be observed in many cases, with a local decrease or absence of lung sliding along the proximal surface of the “wedge sign” (4). Vascular characteristics identified on color Doppler imaging included a “vascular sign*”* described in the human literature (4) which was found in the right cranial lung lobe (Figure 2) within two basket-shaped consolidations. The “*vascular sign*” is believed to result from the occlusion of a vessel by embolic material (4, 24, 25). Abrupt cessation of blood flow at the “tip” of the consolidation can be seen using color Doppler. Human lung ultrasound guidelines suggest finding even one consolidation with a vascular sign is suggestive of PTE (4). The findings of a “wedge sign” and “vascular sign” in this case report prompted consideration of PTE as a differential diagnosis (4, 9) (Figure 2).

A preliminary diagnosis of acute PTE was made based on echocardiographic findings of severe PHT and LUS findings of “wedge signs” which contained a “vascular sign.” Biomarker testing through D-dimer and C-reactive protein (CRP) assays was performed to further support or refute a pro-coagulant and/or inflammatory state. The D-dimer assay measured 0.6 μg/mL (reference values <0.3 μg/mL), while CRP was 32.8 mg/L (reference values <20 mg/mL), which was suggestive of a hypercoagulable state and pneumonia, respectively. A heartworm rapid antigen test was negative.

The dog was treated with enoxaparin (0.8 mg/kg TID for 3 days, tapered to SID for 6 days), rivaroxaban (2.5 mg/kg SID) (29), sildenafil (2 mg/kg TID), pimobendan (0.25 mg/kg BID) (12), torasemide (0.1 mg/kg SID), and enrofloxacin (5 mg SID). After 3 days, a response to treatment was seen, the overall condition of the patient improved, and the dog was discharged to the owner. Diuretics were started by the admitting veterinarian on the first day and continued until recheck examination at day 6 due to the presence of B-lines and suspicion of PHT (30–32). The theophylline and antibiotics were continued because bacterial-associated aspiration pneumonia could not be ruled out based on the presence of leukocytosis, a ventral alveolar pattern on TXR, consolidations and B-lines on LUS, and elevated CRP levels.

**Figure 3:**
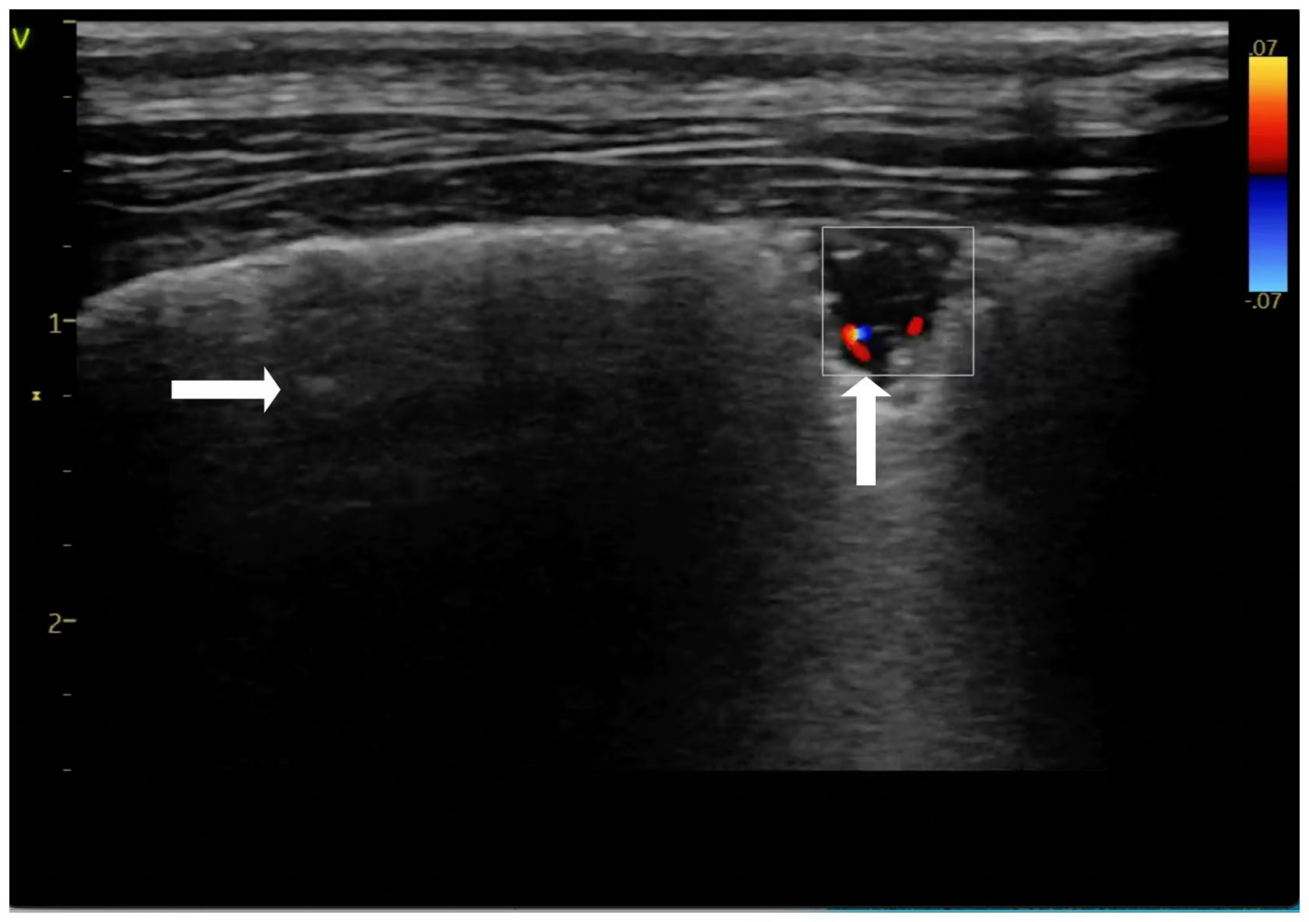
Color doppler imaging of the triangular lung consolidation “wedge sign”, demonstrating recanalization of the consolidation over time (↑), (→) spared areas. Performed on day 6-recheck. Thyroid presets.

**Figure 4:**
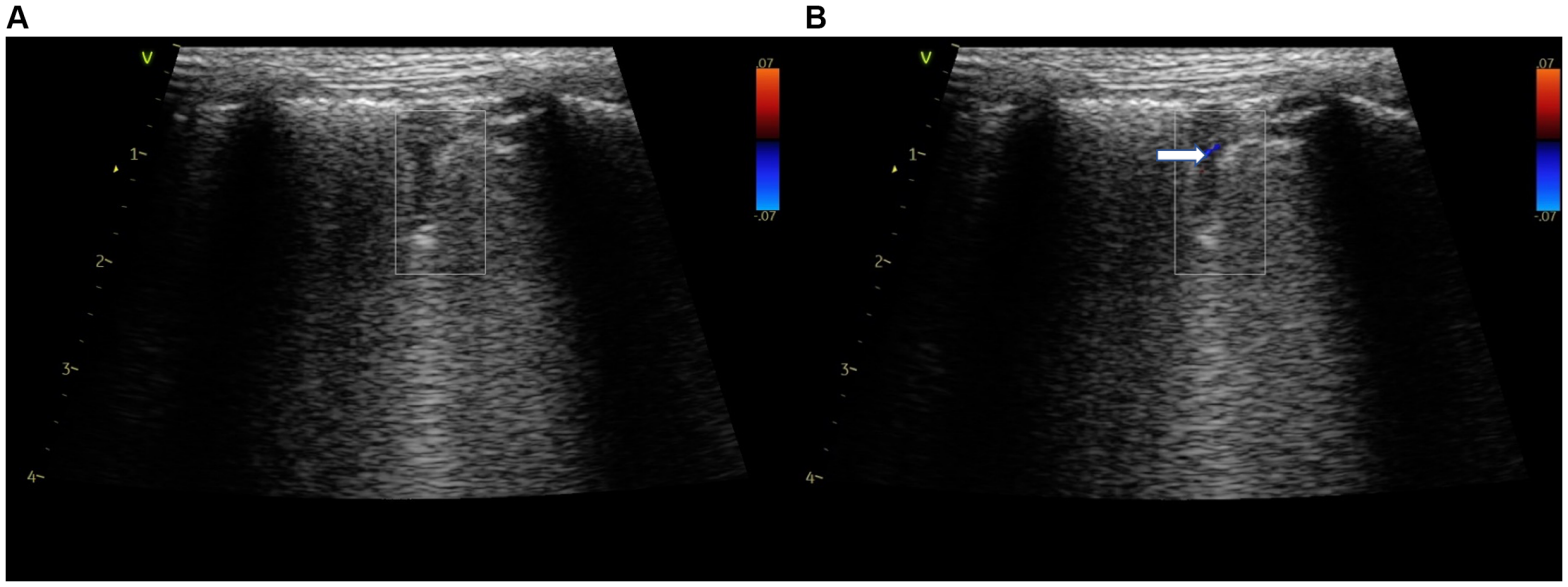
Color doppler still image of a triangular shaped consolidations “wedge sign” with a no flow, long hypoechogenic consolidation **(A)**. Early recanalization of the consolidation using color doppler was seen 6 days following initiation of therapy (→) **(B)**. Lung presets.

Echocardiography, LUS examination, CBC, biochemistry panel, D-dimer, and CRP assays were repeated after 6 days of therapy. The D-dimer assay was <0.3 μg/mL of CRP was <20 mg/L, and the leukocyte count was 17.3 G/L. On echocardiographic examination performed by the same operator, there was a reduction in the RVDd and velocity of tricuspid insufficiency. No pericardial fluid was visible. On LUS examination, recanalization (return of blood flow-based on color Doppler) of the wedge-sign consolidations was noted, as well as a reduced numbers of B-lines (Figure 3; Figures 4A,B). The shred sign consolidations, supportive of inflammatory lung disease and located in the caudodorsal lobes, decreased in size, with some changing in appearance to take on a rounder, smaller hypoechoic shape, similar to a nodule sign (Supplementary Figure S4). Treatment was continued with enrofloxacin for an additional 10 days. Sildenafil, rivaroxaban, and theophylline (10 mg/kg BID) were continued. A Baermann fecal examination was performed 10 days after presentation on a pooled fecal sample collected on three consecutive days, which confirmed *Angiostrongylus vasorum* infection (33, 34). The dog was treated with imidacloprid (10 mg/kg) and moxidectin (2,5 mg/kg) readministered at 4 weeks.

A second recheck examination 45 days following admission, performed by the same operator, revealed the luminal diameter of the right atrium and ventricle were within reference limits and that the tricuspid and pulmonary artery valve regurgitation had resolved. Based on pulsed-wave (PW) Doppler imaging, the pulmonary artery profile was considered unremarkable. Treatment with sildenafil, theophylline, and rivaroxaban was discontinued. Repeat LUS examinations revealed occasional isolated B-lines bilaterally without any visible lung consolidations.

## Discussion

Findings from this case report suggest that color Doppler sonography, in addition to the shape of lung consolidations, can help characterize and differentiate lung lesions in dogs, leading to the consideration of PTE and other inflammatory causes of pulmonary pathology as differential diagnoses. Two types of lung consolidation were present in the current case: “wedge signs” without blood flow suggestive of PTE (the “vascular sign”) and non-descript irregular-shaped consolidations with visible bronchograms and preserved blood flow, consistent with a non-specific inflammatory lesion.

LUS interpretation is based on the assessment of artifacts and consolidations. In a normally aerated lung the bat sign, lung sliding, and A-lines should be visible. A-lines are reverberation artifacts arising as an acoustic phenomenon resulting from the reflection of the ultrasound beams from a highly reflective surface (soft tissue-to-air interface, such as the pleural surface) and its multiple reflections between this surface and the footprint of the transducer (35). Several lung surface artifacts were noted in this case including B-lines, C-lines, and I-lines. The most common pathologic artifacts of LUS are B-lines. Occasional B-lines can be found in healthy dogs, but their presence is associated with lung pathology when increased in number. B-lines move with lung sliding, originate from the pleural line, and extend to the far field of the ultrasound image (25, 35). As the linear transducer was used to differentiate B-lines from other comet-tail artifacts in this case report, the imaging depth was increased to 15 cm to confirm that B-lines did not terminate before reaching the far field of the ultrasound image (25). Unlike B-lines, which can occasionally be normal, the detection of C-lines because of their association with lung consolidation, is always considered an abnormal finding. C-lines, which originate and extend from the distal (deep) border of the lung consolidations as opposed to the pleural line (23). I-lines are described in the literature as short vertical “needle-like” artifacts originating from the pleural line and terminating within a few centimeters; their significance is unclear (Figure 2) (35).

Although a triangular “wedge shape” is the classic sonographic consolidation identified with PTE, being identified in approximately 85% of human patients, PTE can also result in oval, rounded, and polygonal-shaped consolidations (4, 10, 23, 36). Several ventrally located LUS consolidations in the current case were suspected to be the result of PTE based on their triangular or basked shape consistent with a “wedge sign.” In a small case series involving two dogs, a “wedge sign” was considered supportive of PTE, although a definitive diagnosis was not confirmed due to a lack of a reference standard (9). The authors hypothesized that the location of the lesion influenced the likelihood of a “wedge sign” being diagnostic of PTE, with “wedge signs” located caudodorsally supporting the diagnosis, while “wedge signs” located ventrally did not. The concern with “wedge signs” located ventrally is that shred signs, which are commonly located ventrally due to pathology such as aspiration pneumonia, could also appear triangular, making it a challenge to differentiate the two. This led Lisciandro et al. to conclude that the “wedge sign” is indistinguishable from the shred and tissue signs when located in gravity-dependent lung regions (9). To further differentiate the wedge signs from shred signs, we used color Doppler sonography of the lung and identified similar findings to what has been reported in the human literature. Evaluation of the vascular characteristics of consolidations using color Doppler sonography of the lung has been used to provide additional diagnostic information in people when lung lesions are suspected to be caused by PTE, but further confirmation is desired. With pulmonary infarction, pulmonary arterial blood flow cannot be detected by color Doppler ultrasound, referred to as consolidation without perfusion, similar to what was found in the current case (Figure 3) (1, 2, 23, 37). Furthermore, a congested thromboembolic vessel may sometimes be identified within the consolidation, referred to as a “vascular sign,” which was also found in our current case (Figure 2) (2, 3, 5).

The identification of these color Doppler LUS findings supports the diagnosis of PTE in people. By contrast, non-“wedge shaped” consolidations in the current case preserved vascular flow, making PTE less likely. Such irregularly shaped consolidations with preserved blood flow and air bronchograms are considered suggestive of inflammatory lesions in people (4). Findings from the current case report suggest that the application of color Doppler sonography of the lung can help differentiate PTE from other causes of consolidation, even when lesions are localized to the ventral lung regions and/or have an atypical non-triangular-shaped appearance. If blood flow within a consolidated lung region is preserved, it argues against PTE, while the finding of a “vascular sign” and interruption or absence of blood flow within a region of consolidations should prompt consideration of PTE.

In the present case, PTE was suspected based on echocardiography, the presence of a McConnell sign, and elevated D-dimers. Further support for the presence of PTE was identified through the detection of “wedge signs,” particularly when they contained “vascular signs.” These findings, together with the absence of active bleeding, were the rationale for providing antithrombotic therapy. By contrast, the dog also had consolidations with preserved blood flow in the form of a tree-like vasculature pattern on color Doppler, as well as elevated CRP levels. It has been reported that elevations in CRP are associated with canine aspiration pneumonia (38), although there are no studies regarding the diagnostic value of CRP in other forms of pneumonia, including parasitic pneumonia, in dogs. However, this case report suggests that the additional use of color Doppler during LUS examination to characterize the vascular findings of consolidations in conjunction with the shape of the lung consolidation may be helpful in differentiating the cause of lung consolidation. It should be remembered that the absence of findings on LUS does not rule out PTE.

The final diagnosis in the current case was *Angiostrongylus vasorum* based on a positive fecal Baermann test and a positive response to antiparasitic treatment. The LUS findings in cases with *Angiostrongylus vasorum* have been described in the literature, consisting of multiple subpleural pulmonary nodules with or without concomitant B-lines (34). When identified, nodules were always bilaterally located, affecting mainly the caudodorsal areas, with a roughly round shape, slightly hypoechoic echogenicity, and clear margins. Similarly, the current case identified consolidations predominantly in the caudodorsal regions. However, in contrast to the prior report, consolidations in the current case had multiple bronchial findings present, including static and dynamic air bronchograms as well as fluid bronchograms, which were not reported by Venco et al., although retrospectively assessing the images published in the Venco study, it appears some consolidations do contain air bronchograms (34). A significant difference in the consolidations between the two studies is the shape of the lesions: irregular vs. round-shaped. As the classification of consolidations is not standardized in veterinary medicine, it is possible that subjective differences in what is considered round-, irregular-, or basket-shaped may be different between the operators classifying the lesions in the two articles. It is also possible that some of the round-shaped lesions described by Venco were in fact basket-shaped, and therefore may have been caused by PTE, particularly if bronchograms were absent. It is also possible that the timing of assessment between the two studies was different and that the acute imaging of *Angiostrongylus vasorum* may reveal irregular-shaped consolidations in the caudodorsal regions, which become smaller, rounder, and more “nodule” like in appearance as they resolve with lung healing. It is therefore possible that the cases scanned by Venco et al. were in the later stages and resembled the consolidation in the current case during the second recheck evaluation compared to the more acute assessment at the time of referral. Round consolidations are referred to as a “nodule sign” in the literature and can correspond to granulomas found on gross and histopathologic examination in cases of *Angiostrongylus vasorum* invasion (39). Nodule signs may also be related to the presence of neoplastic lesions (both primary and metastatic), and neoplasia should be included in the differential diagnosis of such patients. However, in the presented case, the “nodule sign” evolved from a “shred sign,” so it was not considered neoplastic. Furthermore, neoplasia was considered unlikely given the dog’s young age and lack of interstitial lesions on TXR performed prior to LUS.

Evaluation of the lungs in the current case was performed with a linear probe in contrast to a microconvex probe. A linear probe was chosen in the current report as a higher frequency was preferred for the characterization of any consolidations identified. Based on the recommendations from human medicine, the linear probe may be preferred for pleural and subpleural consolidation assessment (25, 40). However, it should be kept in mind that the microconvex probe is often recommended for vertical lung surface artifact analysis. However, by increasing the depth of the image to 15 cm with the linear probe, the ability of the operator to differentiate vertical artifacts arising from the pleural line (e.g., B-, Z-, or I-lines), and to confirm that B-lines are true B-lines can be achieved by assessing if the vertical artifact reaches the far field and does or does not move with the phases of respiration.

A general disadvantage of LUS is that lesions that fail to reach the lung periphery will not be detected because lung pathology separated from the lung surface by air will be obscured and only reverberation artifacts will be seen. Therefore, LUS cannot rule out PTE, and in human medicine, the finding of a wedge-shaped consolidation with supportive clinical findings is always considered an indication to perform additional tests to further confirm or exclude PTE (4, 10). Further tests may include echocardiography to look for evidence of pulmonary hypertension, non-imaging diagnostics such as thromboelastography or D-dimers, a search for a hypercoagulable state, and the use of color Doppler sonography when reference standard diagnostics are not available.

There are several limitations in this case report. First, we cannot confirm this patient had PTE. Second, there were two types of sonographic consolidation identified with different vascular criteria, so it is possible PHT may have occurred as a result of PTE and/or pneumonia, as both D-dimer and CRP levels were elevated. Furthermore, the dog received multimodal treatment, making it impossible to determine which underlying cause was responsible for the PHT. A reference standard diagnosis was not possible in this case due to a lack of equipment availability and financial constraints on the owner. However, similar to other studies, a clinical standard diagnosis consisting of supportive clinical findings, echocardiography findings, D-dimer results, the presence of a potentially hypercoagulable disease, and color Doppler sonography of the lungs was used. Although LUS can be beneficial as a screening method, it must be considered with the clinical context of the patient and align with the clinical examination and other imaging and non-imaging diagnostic tests.

A general drawback of color Doppler sonography for LUS is that motion artifacts as a result of respirations may interfere with the quality of color Doppler sonography. Good ultrasound settings and Doppler velocity scale adjustments are important to visualize small vessels within lung consolidations. For less experienced operators, Doppler analysis can be difficult to perform. Depending on the availability of equipment in an emergency setting, point-of-care ultrasound machines may have limited Doppler functionality and lower image quality, which may limit their ability to detect PTE. Nevertheless, lung consolidation should be evaluated for vascularity whenever possible. If the patient is experiencing severe dyspnea or panting, LUS can be performed after stabilization of the patient and sedation is administered. Sedatives should not directly influence the results of the examination, but LUS should be performed as soon as possible after achieving sedation to avoid atelectasis, which may occur because of prolonged lateral recumbency.

## Conclusion

In addition to using B-mode LUS to characterize the shape and parenchymal appearance of consolidations, the addition of color Doppler sonography to assess their vascular characteristics can provide supportive information on potential etiologies. Color Doppler may also help guide patient management by supporting a diagnosis of PTE when a hypercoagulable condition is identified and the patient presents with additional clinical findings that further support the diagnosis.

## Data availability statement

The raw data supporting the conclusions of this article will be made available by the authors, without undue reservation.

## Ethics statement

Ethical approval was not required for studies involving animals in accordance with the local legislation and institutional requirements because it is a case report not a clinical trial. Written informed consent was obtained from the owners for the participation of their animal in this study.

## Author contributions

KK: Conceptualization, Writing – original draft, Investigation, Visualization. MG: Supervision, Writing – review & editing. SB: Supervision, Writing – review & editing.

## Supplementary material

Supplementary Figure S1 Thoracic radiographs taken at the time of presentation. **(A)** Dorsoventral, **(B)** right lateral. A bilateral bronchial pattern in the caudal lung regions and a cranial alveolar pattern, which was more prominent on the right, can be seen.

Supplementary Figure S2 Right parasternal short axis still image. The right ventricle (↓) is enlarged, with the flattening of the interventricular septum (→), which suggests pulmonary hypertension. Performed on presentation.

Supplementary Figure S3 Color doppler still image of an irregularly shaped consolidation where the branched vascular structures (appearing tree-like) can be visualized, confirming preserved blood flow within the consolidation. Lung presets. Performed on presentation.

Supplementary Figure S4 Still lung ultrasound image of a round-shaped consolidation referred to as “nodule sign”. Performed at recheck on day 6. Thyroid presets.
