# Supplementary figures and images for: Case report: Application of color Doppler sonography for the assessment of pulmonary consolidations in a dog
Source: Front Vet Sci. 2023 Dec 8;10:1275929. doi: 10.3389/fvets.2023.1275929 (PMC10752369; doi:10.3389/fvets.2023.1275929)

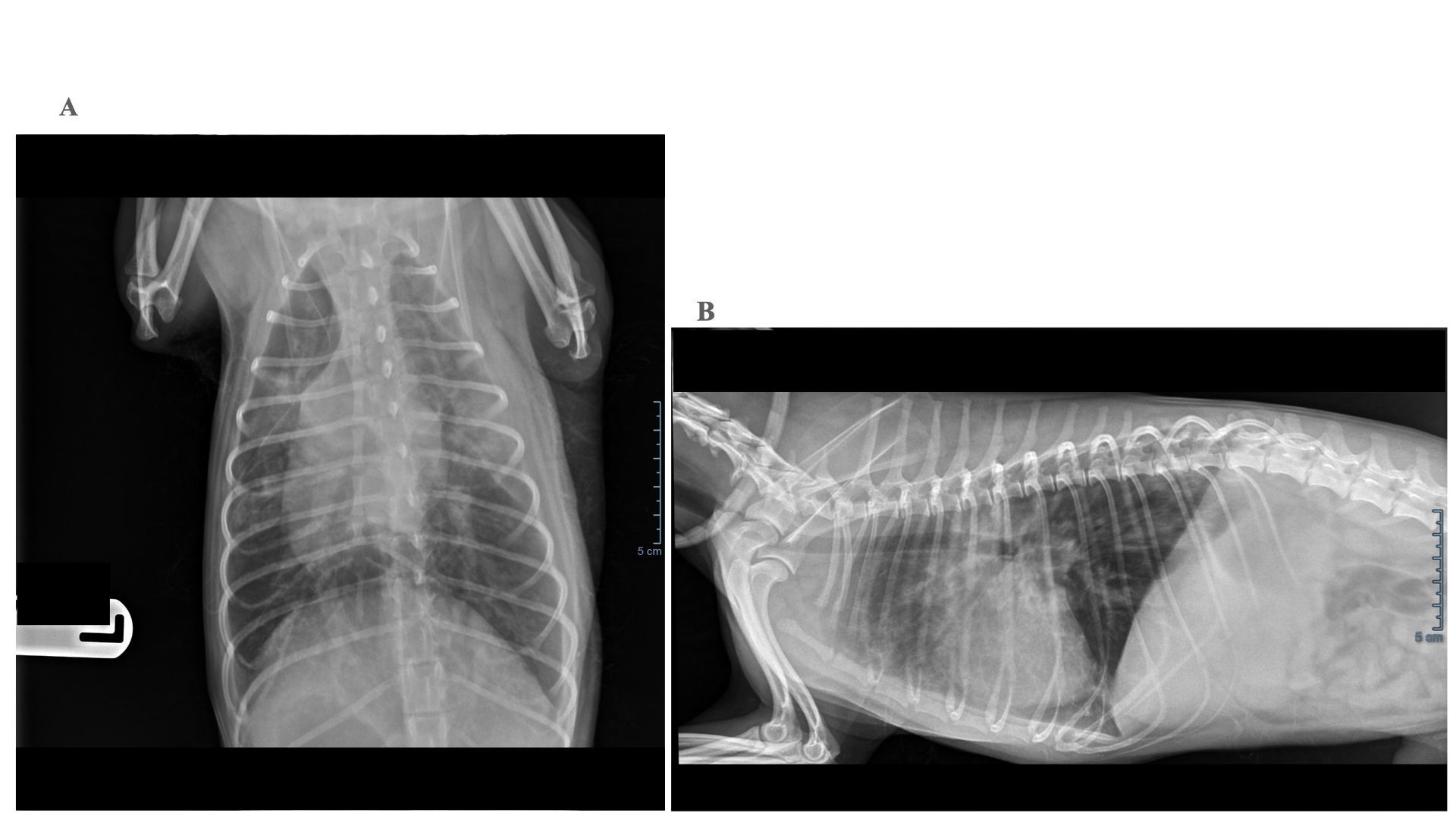

Supplement: Supplementary Figure S1 — Thoracic radiographs taken at the time of presentation. (A) Dorsoventral, (B) right lateral. A bilateral bronchial pattern in the caudal lung regions and a cranial alveolar pattern, which was more prominent on the right, can be seen. [file Image_1.tiff]

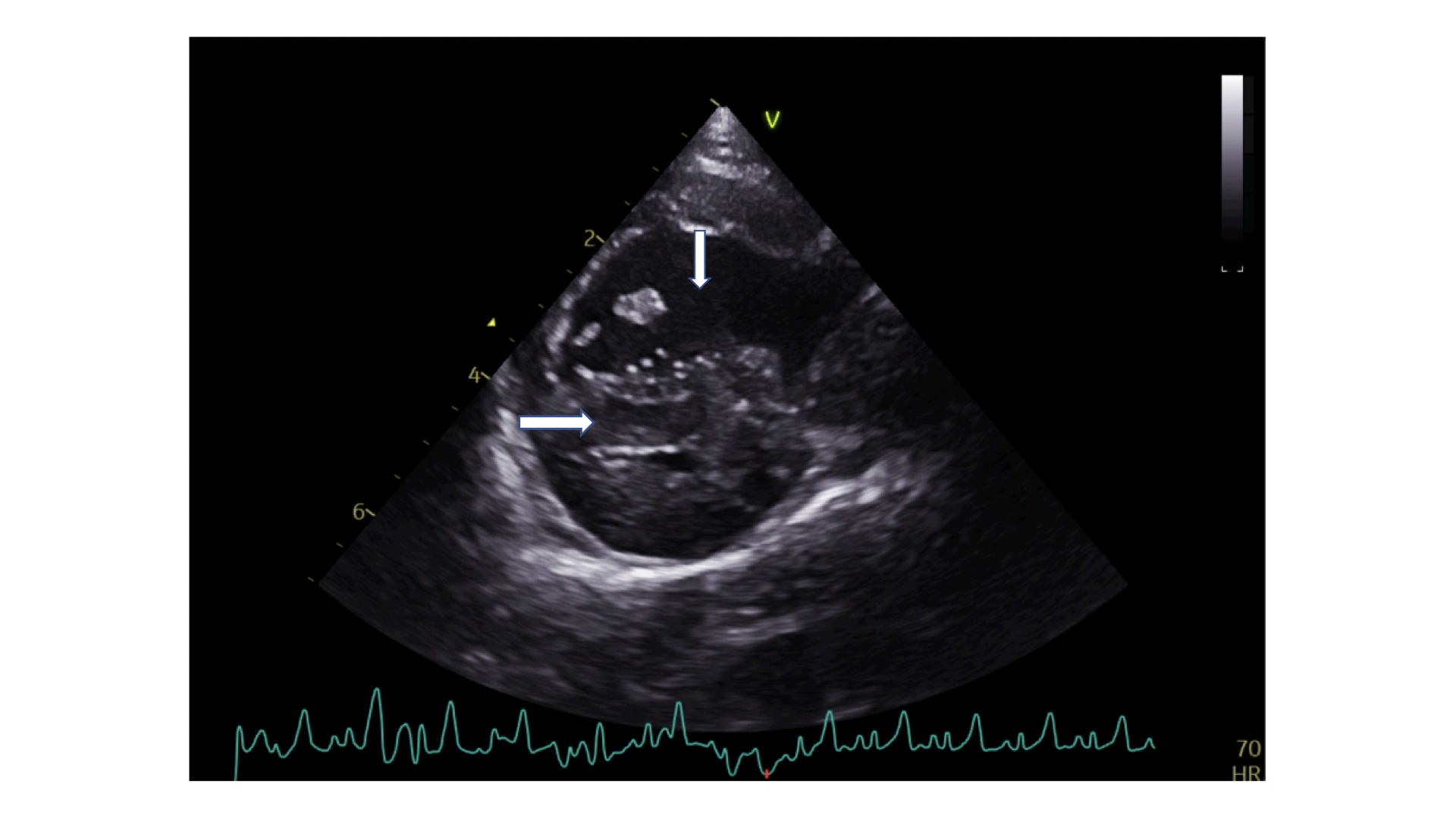

Supplement: Supplementary Figure S2 — Right parasternal short axis still image. The right ventricle (↓) is enlarged, with the flattening of the interventricular septum (→), which suggests pulmonary hypertension. Performed on presentation. [file Image_2.tiff]

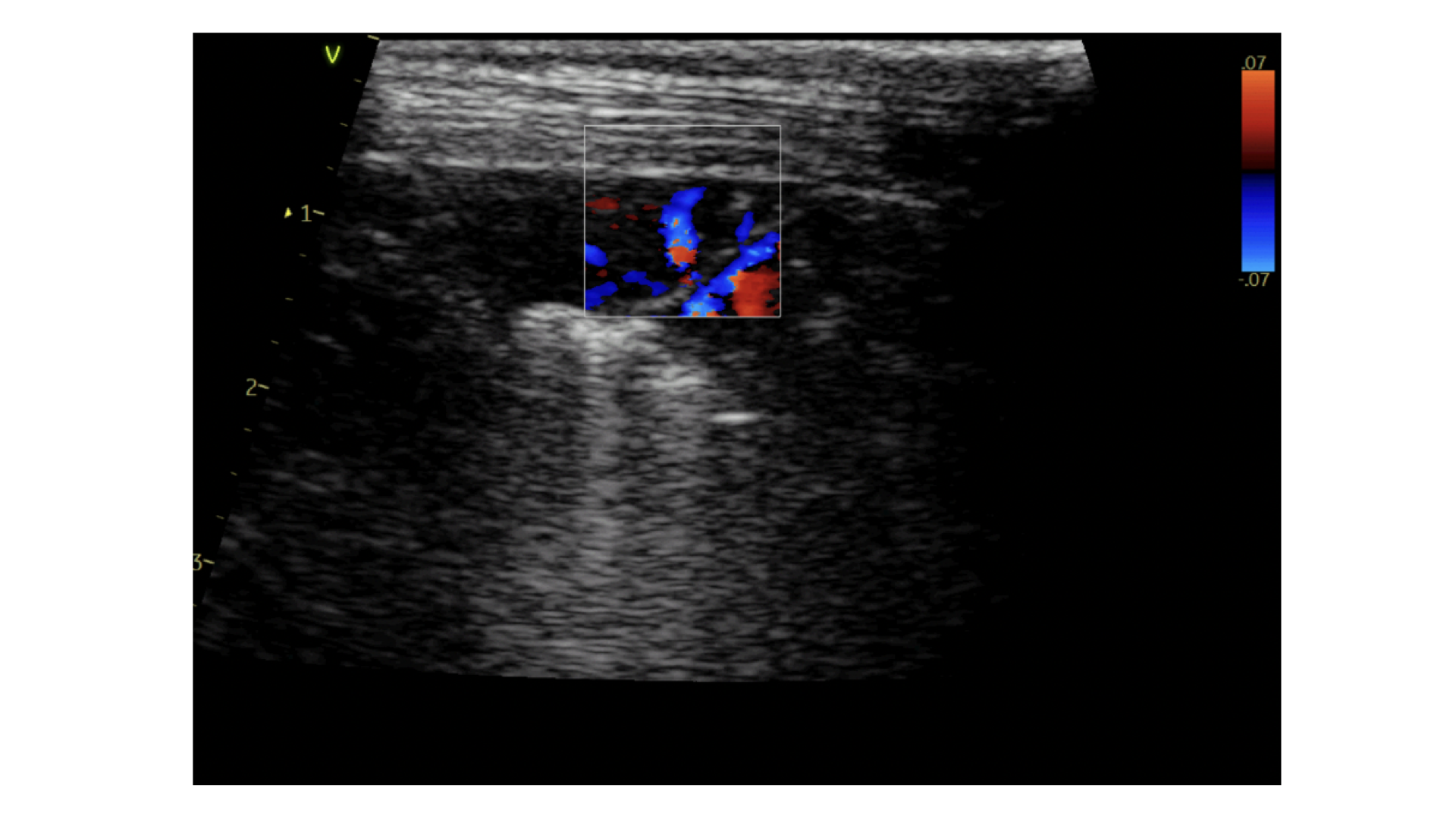

Supplement: Supplementary Figure S3 — Color doppler still image of an irregularly shaped consolidation where the branched vascular structures (appearing tree-like) can be visualized, confirming preserved blood flow within the consolidation. Lung presets. Performed on presentation. [file Image_3.tiff]

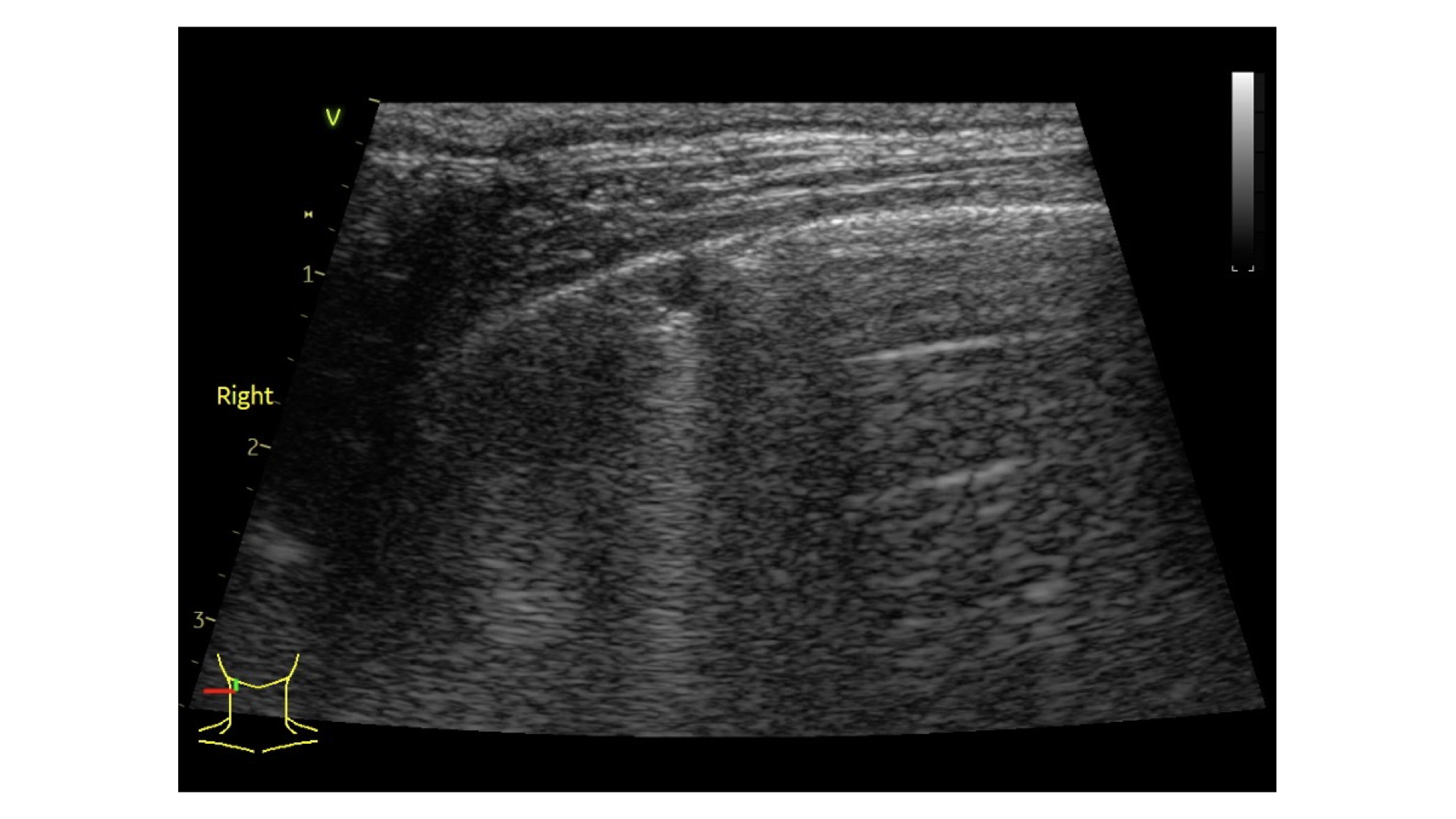

Supplement: Supplementary Figure S4 — Still lung ultrasound image of a round-shaped consolidation referred to as “nodule sign”. Performed at recheck on day 6. Thyroid presets. [file Image_4.TIFF]
